# Gallstone Ileus Secondary to Cholecystoduodenal Fistula: A Case Report and Review of Surgical Management

**DOI:** 10.1155/cris/7852699

**Published:** 2026-04-30

**Authors:** Oluwaseun Adeyemi, Mitsu Patel, Elias Shamoun, Cory Cort, Charles Franco

**Affiliations:** ^1^ Department of Surgery, Rutgers Robert Wood Johnson Medical School, New Brunswick, New Jersey, USA, rutgers.edu; ^2^ Department of Psychiatry, Jersey Shore University Medical Center, Neptune, New Jersey, USA, jerseyshoreuniversitymedicalcenter.com; ^3^ Department of Medicine, St. George University Medical School, True Blue, St. George, Grenada; ^4^ Department of Surgery, New York Medical College, New York, USA, nymc.edu; ^5^ Department of Surgery, Saint Peters University Hospital, New Brunswick, New Jersey, USA

## Abstract

Gallstone ileus is a rare cause of small bowel obstruction (SBO). It occurs when a gallstone passes through a cholecystoenteric fistula and obstructs the bowel. This case report aims to share our experience in managing an uncommon cause of SBO and to discuss available surgical management options, including the rationale for a staged approach in patients with significant comorbidities. We describe a 63‐year‐old woman with a history of hypertension, diabetes, gallstones, and uterine cancer treated with hysterectomy and radiation therapy, who presented with right upper quadrant pain and vomiting and subsequently developed signs of SBO. A computerized tomography scan of the abdomen and pelvis revealed a cholecystoduodenal fistula with evidence of a stone in the distal ileum. She subsequently underwent emergency enterolithotomy, successfully removing the stone, and was scheduled for a cholecystectomy at a later date to reduce her surgical risk. We discuss the three approaches to the surgical management of gallstone ileus—enterolithotomy alone, one‐stage surgery (enterolithotomy + cholecystectomy with fistula repair), and two‐stage surgery (enterolithotomy and delayed cholecystectomy with fistula repair).

## 1. Introduction

Gallstone ileus, a rare cause of small bowel obstruction (SBO) [1–3], occurs when a gallstone passes into the gastrointestinal tract and lodges within the bowel lumen [1, 4]. Typically associated with cholecystitis [4, 5], gallstone ileus most commonly occurs when a fistula forms between the gallbladder and adjacent bowel, usually the duodenum, allowing stones to migrate into the gastrointestinal tract [1, 4]. Although most cases of SBO are caused by adhesions, hernias, or neoplasms [6], gallstone‐related SBO represents an uncommon yet significant surgical challenge [1–3]. The clinical presentation is often nonspecific, with symptoms of abdominal pain, nausea, vomiting, and bloating, making diagnosis difficult [2, 7]. Moreover, imaging may fail to reveal the obstructing stone, delaying appropriate management [8]. This case report presents a rare instance of gallstone‐induced SBO in a 63‐year‐old patient, discussing the diagnostic and surgical approaches required to address this condition. It emphasizes the importance of early recognition and highlights the need for heightened awareness of this etiology, particularly in patients with a history of cholelithiasis who present with atypical SBO.

## 2. Case Report

A 63‐year‐old female presented to the emergency department with a 30 h history of worsening colicky pain in the right upper quadrant of the abdomen. The pain began after consuming a spicy meal and was associated with nausea and episodes of nonbilious, nonbloody vomiting. She denied bloating, and her last bowel movement occurred 36 h before symptom onset. The patient reported experiencing similar episodes of pain over the preceding 12 months that resolved spontaneously. The most recent episode occurred 2 months prior to presentation, at which time she was diagnosed with symptomatic biliary colic. She opted against surgical intervention due to the resolution of symptoms. Her medical history was significant for well‐controlled hypertension and diabetes. Additionally, she is a uterine cancer survivor, having undergone a hysterectomy and radiation therapy.

**Figure 1:**
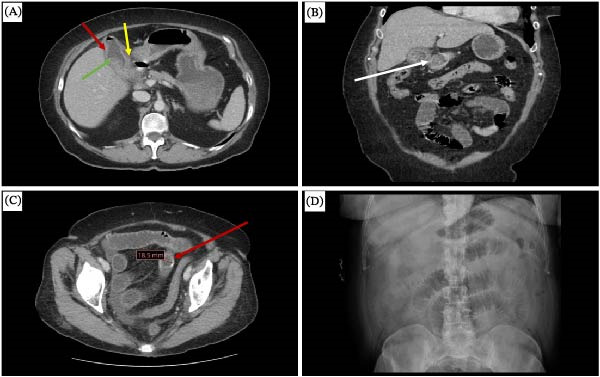
Radiological images showing (A) axial cut showing the loss of fat plane between the gall bladder and the duodenum (yellow arrow), pericholecystic fluid (red arrow), and gallstones (green arrow). (B) Coronal view showing the loss of fat plane between the gall bladder and the duodenum (white arrow). (C) Axial view showing the calcified stone in the distal ileum (red arrow). (D) Distended bowel loop of an abdominal X‐ray taken on day 2 of admission.

On examination, the patient was alert, oriented, and in painful distress. The abdomen was protuberant but not distended. She exhibited tenderness in the right upper quadrant, accompanied by a positive Murphy’s sign. There was no rebound tenderness or evidence of peritonitis, and bowel sounds were normal. Laboratory tests revealed a normal white blood cell count, accompanied by a left shift in neutrophils (Table 1). Electrolytes and liver enzyme levels were within normal limits. A contrast‐enhanced computed tomography (CT) scan of the abdomen and pelvis revealed a moderately thick‐walled gallbladder with surrounding pericholecystic fluid. The gallbladder contained stones and foci of gas, with a loss of the fat plane between the gallbladder and the adjacent duodenum (Figure 1A,B). No biliary dilatation or pneumobilia was observed. Additionally, a rounded, rim‐calcified structure consistent with a gallstone was identified in the mid‐ileum, measuring 18.5 mm (Figure 1C).

**Table 1:** Preoperative and postoperative laboratory values.

Laboratory	Reference ranges	Admission day 1	Admission day 2	Postoperative day 1
Hemoglobin (g/dL)	12.0–16.0	14.5	13.3	13.8
WBC (10^3^/mm^3^)	4.0–11.0	10.1	8.9	8.2
Platelets (10^3^/mm^3^)	150–400	353	307	325
Neutrophil (%)	37.0–75.0	**87.8**	**91.0**	73.9
Lymphocyte (%)	12.0–50.0	8.2	4.0	15.7
Sodium	136–145	138	135	137
Potassium	3.5–5.1	5.0	4.1	3.8
Chloride	99–112	103	105	106
Bicarbonate	21–33	25	28	25
BUN (mg/dL)	9–28	13	9	7.0
Creatinine	0.52–1.04	0.44	0.54	0.49
ALT	0–35	25	12	16
AST	14–36	31	19	23
ALP	53–141	127	58	95
Total bilirubin	0.1–1.2	0.7	0.6	0.8
PTT	27.2–35.7	—	29.9	—
PT	10.4–13.7	—	12.3	—
INR	0.89–1.11	—	1.11	—

*Note:* Values in bold represent elevated values.

Abbreviations: ALP, alkaline phosphatase; ALT, alanine transaminase; AST, aspartate transaminase; BUN, blood urea nitrogen; INR, international normalized ratio; PT, prothrombin time; PTT, partial thromboplastin time; WBC, white blood cell.

These findings were most consistent with cholecystoduodenal fistula and gallstone ileus, accompanied by gallbladder wall thickening and acute inflammation. Given the patient’s clinical presentation, the radiological evidence of the fistula, and a stone that is less than the 2.5 cm impaction‐prone cutoff [8], a conservative approach was adopted with the hope that the stone may migrate into the more spacious colon. We discussed the possibility of having surgery if the expectant management fails. She was placed on nil per oris, started on intravenous fluids at maintenance, administered intravenous analgesics, and scheduled for serial abdominal examinations to monitor for distension and high‐pitched bowel sounds as a clinical marker of obstruction.

On the second day of admission, the patient’s abdominal pain persisted. She reported no nausea or vomiting, and she did not have any bowel movements. She, however, reported some feeling of bloating, and this was confirmed during serial abdominal examination. A supine abdominal X‐ray was done, which reported mild gaseous distention of multiple small bowel loops throughout the abdomen (Figure 1D). Based on the development of SBO, the diagnosis was gallstone ileus. Given the imminent failure of conservative management, the decision was to proceed with an emergency exploratory laparotomy and enterolithotomy. Cholecystectomy was to be deferred to a later date due to concerns that the patient might not tolerate the physiological stress of a single‐stage procedure.

**Figure 2:**
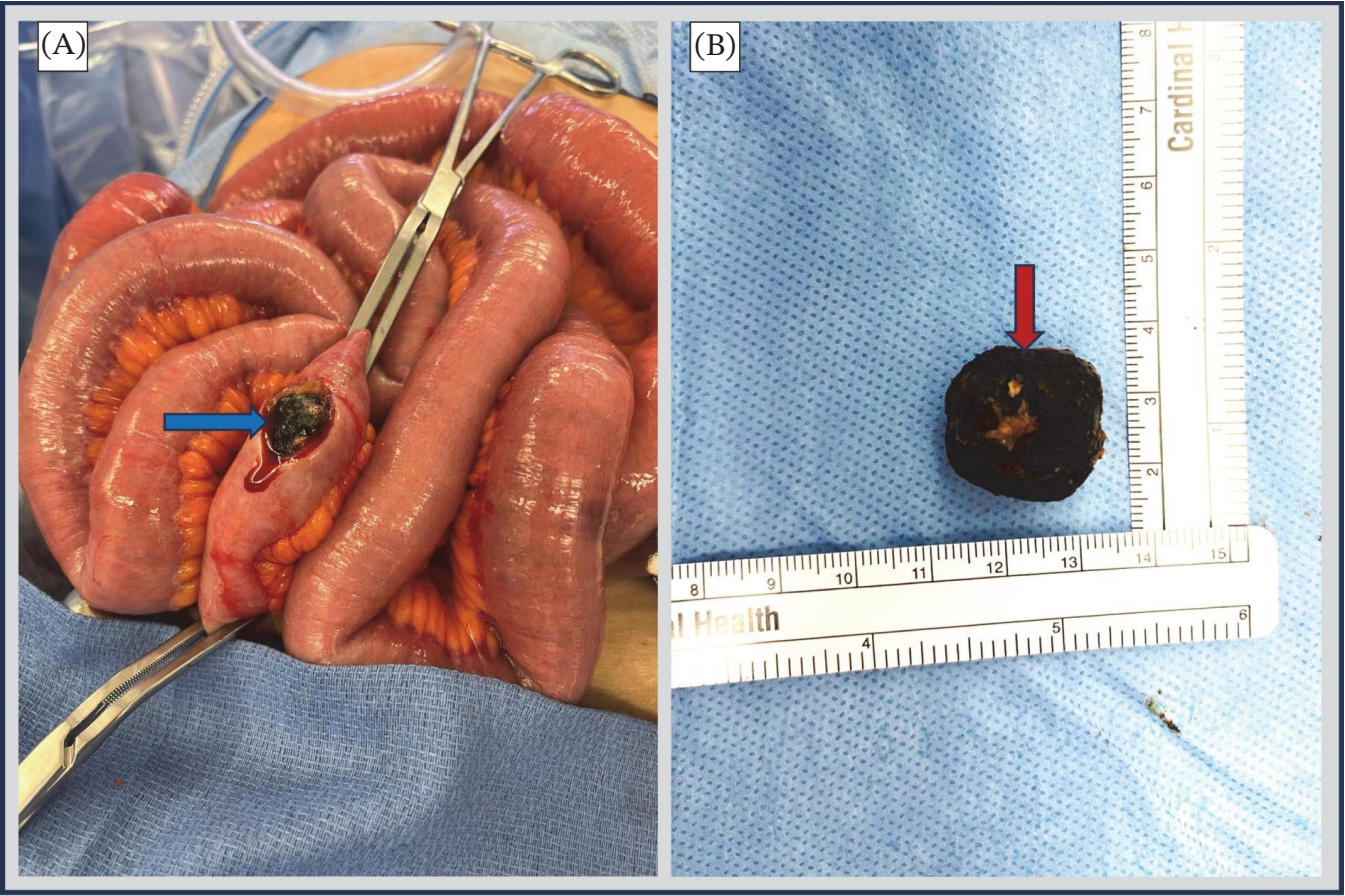
Photos of the (A) cholesterol gallstone being delivered via an enterostomy, and the (B) extracted cholesterol gallstone measuring approximately 20 mm × 20 mm. The blue arrow in (A) and the red arrow in (B) identify the cholesterol gallstone.

The patient underwent an exploratory laparotomy with enterolithotomy. A midline incision was made and deepened through the subcutaneous tissues to the fascia, allowing entry into the peritoneal cavity. Extensive adhesions were encountered and lysed, enabling clear visualization of the intra‐abdominal organs. A systematic bowel examination, beginning at the ileocecal junction, revealed a large gallstone within the distal ileum. The small bowel was moderately dilated up to the distal ileum, where a single large gallstone was identified as the transition point causing obstruction (Figure 2A). No additional stones were identified on further systematic examination of the small bowel. A longitudinal enterotomy was performed on the antimesenteric border directly over the impacted stone, which was successfully extracted (Figure 2B). The enterotomy was closed in a transverse orientation to reduce the risk of luminal narrowing. The bowel was returned to the abdominal cavity, the fascia was closed with continuous absorbable stitches, and the skin was closed with staples. The postoperative course was uncomplicated. Bowel function returned on postoperative day two, and the diet was advanced as tolerated. Postoperative laboratory values were within normal limits, with no elevation in liver enzymes (Table 1). The patient was discharged on postoperative day three with instructions to follow up for an elective cholecystectomy.

## 3. Discussion

Gallstone ileus is a rare complication, but when it occurs, the obstructing stone typically lodges in the narrowest sections of the bowel, most commonly in the ileum, due to its smaller lumen [4]. In patients with a history of prior abdominal or pelvic surgery or cancer‐related radiation therapy, postoperative adhesions are a more common cause of SBO [9]. However, in this case, CT demonstrated a cholecystoduodenal fistula and an ectopic gallstone within the distal ileum, confirming gallstone ileus as the underlying etiology despite the presence of other plausible causes.

The failure of conservative management indicated the need for a surgical intervention. Although gallstone ileus is generally associated with stones larger than 2.5 cm, in our case, a stone ~2 cm in size caused the obstruction. This is likely attributable to chronic radiation enteritis, which reduces the caliber of the small intestine. We opted for a two‐stage procedure because of her comorbidities, including hypertension, diabetes, and a history of uterine cancer and chronic effects of radiation therapy, which increased her risk of complications during a more extensive, one‐stage surgery. The decision to initially address the obstruction through enterolithotomy alone allowed her to recover from the immediate crisis and stabilize, thereby minimizing the perioperative risks associated with a prolonged surgery.

Surgical management of gallstone ileus offers three approaches, each tailored to the patient’s condition and the location of the obstruction: (1) enterolithotomy alone, (2) one‐stage procedure, (3) two‐stage procedure [7]. The most common approach is enterolithotomy, which involves isolating and removing the obstructing gallstone through an incision in the bowel without additional surgery [10]. This method is typically favored in patients with significant comorbidities, as it avoids prolonged operative time and reduces the risk associated with more extensive procedures. There is a possibility for a natural closure of the fistula, and this may occur in more than 60% of cases [11]. By focusing solely on relieving the obstruction, enterolithotomy minimizes the immediate surgical burden, making it particularly suitable for older or hemodynamically unstable patients [12–14]. Additionally, it is a faster procedure, which can be critical in emergent settings where rapid intervention is required to alleviate symptoms and prevent further complications [12–14]. However, enterolithotomy does not address the underlying pathology, such as the cholecystoduodenal fistula or gallbladder disease, leaving patients at risk for recurrence of gallstone ileus, ongoing biliary symptoms, or subsequent complications like cholangitis or cholecystitis.

Alternatively, a more comprehensive approach involves a one‐stage procedure, combining enterolithotomy with cholecystectomy and fistula repair [14, 15]. This approach addresses both the immediate bowel obstruction and the underlying pathology, including the gallbladder and fistula, in a single operation [12, 15]. Resolving the source of the gallstone and repairing the fistula reduces the risk of recurrent gallstone ileus and complications such as recurrent fistula formation, biliary sepsis, or cholecystitis. However, the one‐stage procedure is associated with increased operative time and complexity, which may elevate the risk of perioperative complications, particularly in elderly or medically frail patients [14, 15]. It is also associated with increased length of hospital stay and mortality [16]. The extensive nature of this approach requires careful patient selection, as it demands a stable cardiovascular and respiratory status to tolerate the prolonged surgery. Despite its risks, the one‐stage procedure can be advantageous in fit patients where the potential for future complications warrants definitive treatment during the initial intervention.

The two‐stage procedure is another approach for managing gallstone ileus, in which the initial operation consists of enterolithotomy to relieve the bowel obstruction. A second procedure, typically performed after the patient has stabilized, involves cholecystectomy with consideration of fistula repair if the fistula does not close spontaneously [7, 15]. This staged approach is beneficial in patients who are considered high‐risk for the more extensive one‐stage procedure due to age, comorbidities, or frailty. It minimizes operative time and perioperative stress during the initial surgery, thereby reducing the risk of complications such as bleeding, infection, and cardiovascular or respiratory strain. One of the key advantages of the two‐stage approach is that it allows the patient time to recover from the first surgery, thereby improving their overall fitness for a second, more complex procedure [3]. Additionally, a two‐stage procedure allows evaluation of the patient’s response to the initial surgery and confirmation of bowel obstruction resolution before proceeding with further interventions [7, 15]. However, the two‐stage approach is not without drawbacks. It requires two separate surgeries, each carrying its own risks, including those associated with anesthesia and postoperative recovery. The delay in performing the cholecystectomy and fistula repair increases the risk of recurrent gallstone ileus [17, 18] or bowel perforation [19, 20]. Furthermore, the two‐stage approach may lead to a longer overall recovery period and an increased psychological burden due to the need for two separate operations.

The management of gallstone ileus has important implications for patients, surgeons, and the healthcare system. For the patient, early recognition can significantly improve outcomes and reduce the risk of complications, leading to better recovery. For the surgeon, a careful assessment of the patient’s health status and comorbidities is essential in determining the appropriate surgical approach, whether it be a one‐stage or staged procedure, to minimize operative risk and ensure successful outcomes. For the healthcare system, efficient diagnosis and management are crucial for optimizing resource utilization, reducing hospital stay durations, and improving the quality of care for patients with complex medical conditions.

## 4. Conclusion

Gallstone ileus is an uncommon but important cause of SBO, particularly in patients with a history of gallstone disease. Early recognition and appropriate imaging are essential for timely diagnosis. Surgical management should be individualized based on patient comorbidities and physiological reserve. In high‐risk patients, enterolithotomy alone with consideration of staged biliary surgery represents a safe and effective approach for relieving obstruction while minimizing operative risk.

## Author Contributions

**Oluwaseun Adeyemi**: study conception and design. **Oluwaseun Adeyemi and Charles Franco**: acquired data and images. **Oluwaseun Adeyemi**, **Mitsu Patel**, **Elias Shamoun, and Cory Cort**: drafted the manuscript. **Oluwaseun Adeyemi**, **Elias Shamoun**, **Mitsu Patel**, **Charles Franco, and Cory Cort**: critical review and revision of the final manuscript.

## Acknowledgments

We acknowledge all other residents and attendings in the Department of Medicine, Anesthesia, and the Department of Radiology who were involved in the care of this patient.

## Funding

The authors received no financial support for the research, authorship, and/or publication of this article.

## Ethics Statement

Our institution does not require ethical approval for reporting individual cases or case series.

## Consent

Written informed consent was obtained for the publication for the information included in this article. Written informed consent for publication was provided by the participant.

## Conflicts of Interest

The authors declare no conflicts of interest.

## Data Availability

Data sharing does not apply to this article as no new data were created or analyzed in this study.
